# Epidemiology and Surveillance of Influenza Viruses in Uganda between 2008 and 2014

**DOI:** 10.1371/journal.pone.0164861

**Published:** 2016-10-18

**Authors:** Fred Wabwire-Mangen, Derrick E. Mimbe, Bernard Erima, Edison A. Mworozi, Monica Millard, Hannah Kibuuka, Luswa Lukwago, Josephine Bwogi, Jocelyn Kiconco, Titus Tugume, Sophia Mulei, Christine Ikomera, Sharon Tsui, Stephen Malinzi, Simon Kasasa, Rodney Coldren, Denis K. Byarugaba

**Affiliations:** 1 Makerere University, School of Public Health, College of Health Sciences, Kampala, Uganda; 2 Makerere University Walter Reed Project, Kampala, Uganda; 3 Ministry of Health, Kampala, Uganda; 4 Uganda Virus Research Institute, Entebbe, Uganda; 5 John Hopkins University, Baltimore, Maryland, United States of America; 6 United States Army Medical Research Directorate—Kenya, Nairobi, Kenya; 7 Makerere University, College of Veterinary Medicine, Animal Resources and Biosecurity, Kampala, Uganda; George Mason University, UNITED STATES

## Abstract

**Introduction:**

Influenza surveillance was conducted in Uganda from October 2008 to December 2014 to identify and understand the epidemiology of circulating influenza strains in out-patient clinic attendees with influenza-like illness and inform control strategies.

**Methodology:**

Surveillance was conducted at five hospital-based sentinel sites. Nasopharyngeal and/or oropharyngeal samples, epidemiological and clinical data were collected from enrolled patients. Real-time reverse transcription polymerase chain reaction (RT-PCR) was performed to identify and subtype influenza strains. Data were double-entered into an Epi Info 3.5.3 database and exported to STATA 13.0 software for analysis.

**Results:**

Of the 6,628 patient samples tested, influenza virus infection was detected in 10.4% (n = 687/6,628) of the specimens. Several trends were observed: influenza circulates throughout the year with two peaks; the major one from September to November and a minor one from March to June. The predominant strains of influenza varied over the years: Seasonal Influenza A(H3) virus was predominant from 2008 to 2009 and from 2012 to 2014; Influenza A(H1N1)pdm01 was dominant in 2010; and Influenza B virus was dominant in 2011. The peaks generally coincided with times of higher humidity, lower temperature, and higher rainfall.

**Conclusion:**

Influenza circulated throughout the year in Uganda with two major peaks of outbreaks with similar strains circulating elsewhere in the region. Data on the circulating strains of influenza and its patterns of occurrence provided critical insights to informing the design and timing of influenza vaccines for influenza prevention in tropical regions of sub-Saharan Africa.

## Introduction

Influenza can cause severe morbidity and even mortality in patients [1–3] with many cases requiring hospitalization for care and even intensive care for the most severe cases [4–8]. Influenza poses a serious threat to public health nationally and globally because of its unpredictable and rapid mutational capabilities. This is evidenced by the recent emergence of zoonotic strains, which can transmit disease from animals to humans, including influenza A (H1N1) pdm09 [4, 6, 8–10], H7N9 [2, 11, 12], and H5N1 [13–16]. Given influenza viruses’ ability to evolve and cause pandemic outbreaks, it is vital to undertake surveillance to monitor and rapidly detect emergence of these viruses in animals and humans to ensure country preparedness. The monitoring of genetic changes in these viruses is recommended to identify virus strains and inform vaccine selection specific to the needs of the region [17, 18]. The practice of influenza surveillance is in accordance to the International Health Regulation (2015), which calls for surveillance “to prevent, protect against, control, and provide a public health response to the international spread of diseases in ways that are commensurate with and restricted to public health risks, and which avoids unnecessary interference with international traffic and trade”.

Influenza surveillance activities in tropical regions, particularly developing countries in sub-Saharan Africa, have been limited until recently. Previously, the WHO grouped all countries in the Southern hemisphere together and recommended preventive vaccinations that were tailored to countries located in the temperate regions of the Southern hemisphere. However, there is growing evidence that influenza outbreaks vary by climatic conditions and there is a need to tailor preventive interventions accordingly. Temperate countries usually have only one or two influenza outbreaks each year with the most frequent influenza-like illnesses in the winter months; in contrast, tropical countries have influenza circulating all-year round with peaks around the rainy seasons [18]. In view of these differences, the WHO and partners have invested greater resources to monitor influenza in temperate regions of the Southern hemisphere.

A clearer understanding of the epidemiology of influenza and viral characteristics are essential to inform public health experts on vaccine selection and the timing of preventive vaccination for the tropical regions. Prompted by the threats of the H5N1 pandemic in 2005, Makerere University Walter Reed Project (MUWRP) began surveillance of influenza viruses in collaboration with the Ugandan Ministry of Health. The goals of surveillance were two-fold: 1) to set up infrastructure for detection and characterization of influenza viruses in order to provide a rapid response to potential pandemics; and 2) to use long-term surveillance data to elucidate the epidemiology of seasonal influenza and inform vaccination strategies. In 2011, MUWRP reported on the molecular diagnostic methods and whole genome analysis of influenza A/H3N2 [19]. In this paper, we describe the epidemiology of circulating strains of influenza A, including A/H3N2, A/H1N1 pdm09, and the co-infection of H3N2 and H1N1 pdm09, and B from 2008 to 2014.

## Materials and Methods

### Surveillance Sites

Hospital-based sentinel sites were selected through purposive sampling in consultation with the Ugandan Ministry of Health using the following criteria: rural and urban settings, along trade routes, major landing sites for migratory birds and live bird markets. Five government hospitals namely, Mulago National Referral hospital, Jinja and Gulu Regional Referral hospitals, Kayunga and Bugiri District hospitals were identified for influenza surveillance activities. The five selected sites are located in the three main geographical distributions of the country namely, Central (Mulago and Kayunga), Eastern (Jinja and Bugiri) and Northern (Gulu) Uganda.

### Study Population

Any individuals presenting with signs and symptoms of respiratory infections at the outpatient clinic sentinel sites, regardless of sex and age, were eligible for the study screening.

#### Case Definition

The case definition used for Influenza-Like Illness (ILI) in this study included:

1. Fever ≥38° C (axillary); and
2. Cough or sore throat; and
3. Onset of ILI within the past 72 hours (3 days).

All those who did not meet the case definition or were not willing to participate were excluded from the study.

### Study Procedures

#### Enrolment of Participants

Patient enrollment occurred from Monday to Friday during normal working hours (8 am– 5 pm) at the sentinel sites. Patients who presented with ILI symptoms were identified from the outpatient departments waiting area for systematic screening and inclusion into the study by trained clinical interviewer. The clinical interviewer then used a brief screening tool with the list of inclusion criteria to determine study eligibility. Eligible participants were provided with information about the influenza surveillance and invited to participate in the study. Interested and eligible participants underwent an informed consent process in English or the appropriate local language to learn about the study. Willing and eligible participants were asked to provide written consent if 18 years or older, written assent if 8–17 years old, and written consent were obtained from parents or guardians of all minors less than 18 years. Separate informed consent for storage and future use of remaining sample aliquots was obtained from participants. After enrollment, trained clinician interviewers allocated a unique personal identification number (PIN) to each individual study participant, administered a study questionnaire, performed a physical examination, and collected a nasopharyngeal and/or oropharyngeal sample. Finally, study participants were reimbursed for their time.

#### Clinical and Epidemiologic Information

Clinician interviewers were trained to administer a standardized questionnaire using English or the local language the participant best understood. The questionnaire collected the following information from the participant: socio-demographic characteristics, socio-economic activities, history of exposure to birds and animals, clinical symptoms and signs for influenza, and recent history of influenza-like illness. In addition, the clinician interviewers took a medical history and conducted physical examination.

#### Specimen Collection

Nasopharyngeal and oropharyngeal swabs were obtained from study participants who met all the criteria of a positive case definition for ILI. Where it was not possible to obtain both a nasopharyngeal and oropharyngeal swab from a participant, only one of the two, either a nasopharyngeal or oropharyngeal swab was collected. Samples were collected in the privacy of a screened study area. Nasopharyngeal and oropharyngeal swabs were taken using Dacron swabs and placed in 2.0 mLs viral transport medium (VTM). Each cryovial was labeled with the participant’s PIN, wiped with the appropriate disinfectant, temporarily stored in specimen transportation bags placed in cold boxes with ice packs, and later transferred to a dry shipper at temperatures below -150°C.

#### Laboratory Procedures

**Fig 1:**
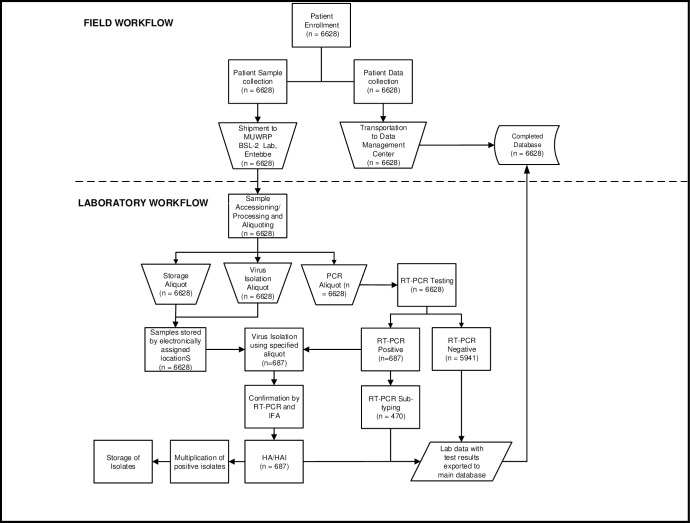
MUWRP Influenza Surveillance Work Flow.

The samples and accompanying data collection forms were transported once a week in sealed envelopes placed in sealed boxes for testing at the Makerere University Walter Reed Project-Emerging Infectious Diseases Laboratory (MUWRP-EIDL) (Fig 1).

#### Specimen Processing

The cryovials containing the nasopharyngeal and oropharyngeal swabs were stored at temperatures between -82°C and -80°C. The cryovials were identified using pre-printed labels with PINs. MUWRP laboratory personnel oversaw the logistics of sample collection and accessioning samples on a routine and timely basis.

#### Influenza A and B Screening

Samples were screened and subtyped using real-time reverse transcription polymerase chain reaction (RT-PCR) as previously described by Byarugaba et al. (2011) [19]. Aliquots of PCR positive samples were inoculated in Madin-Darby canine kidney (MDCK) cell lines for virus isolation and culture and further confirmed using immuno-fluorescence assay and PCR and also Hemagglutination (HA) and Hemagglutination inhibition (HAI) assays (Fig 1). Virus isolates were typed using RT-PCR for the hemagglutination genes and hemagglutination inhibition (HAI) using guinea pig red blood cells, in accordance with CDC protocols and WHO recommendations. A detailed description of the influenza screening processes for influenza A and influenza B viruses can be found in Byarugaba. et al. (2011) and Byarugaba. et al. (2013) respectively [19, 20].

#### Climatic Data

Rainfall (millimeters), relative humidity (percentage), and temperature (degrees Celsius) data disaggregated by geographic sites were obtained from the Uganda National Meteorological Authority for the surveillance period from 2008 to 2014.

### Data Management and Analysis

All data were double entered into Epi Info 3.5.3 (CDC, Atlanta, Georgia) data entry screens to identify data entry errors. Inconsistencies between data entrants were rectified by consulting the hardcopy data forms. The cleaned Epi Info dataset was exported to Stata 13.0 (Stata Corporation, College Station, Texas) for statistical analysis. Eleven participants with incomplete data were excluded from the demographic analysis. Participant demographic characteristics, such as age, sex, and education, were summarized and compared by influenza status and type using Chi-Square and Fisher’s Exact Test. Exploratory data analysis was carried out at monthly, quarterly, and annual time intervals to identify trends over time. Average climatic data were then plotted against the number of influenza cases over the surveillance period to assess the association between influenza peaks and seasonal climatic variation.

### Ethics Statement

The influenza and influenza-like illnesses surveillance study was reviewed and approved by the Makerere University School of Public Health Research and Ethics Committee (Protocol # 020), the Walter Reed Army Institute of Research Institutional Review Board, (Protocol #1436) and the Uganda National Council for Science and Technology (Protocol # HS 377). Eligible and willing participants underwent an informed consent process in English or the local language for the site. All adult participants provided written informed consent. Parents and guardians of minors less than 18 years provided written consent on behalf of their children. In addition, minors between ages 8 to 17 also provided written assent.

## Results

### Demographic Characteristics

A total of 6,628 patients fulfilled the ILI case definition and were enrolled into the study. Eleven individuals were excluded from the demographic analysis because of missing data for sex and age, but all 6,628 patients were included in the rest of the analysis.

Of the remaining participants, 3259 (49.2%) were female and 3366 (50.8%) were male. The majority 5377 (81.2%) were less than 5 years with most between 1 and 4 years old. The median age of the participants was 2 years with an inter-quartile range of 2. Overall, 90.7% of the participants had not received any education and this was expected as the majority of were less than 5 years old. Patients who tested positive for influenza were more likely to be female (52.7%) and aged 1–4 years (56.6%) (Table 1).

**Table 1:** Demographic characteristics of patients in the 5 health facilities.

		**Specimens status**			
Characteristics		Positive	Negative	Total	Chi-square (p-value)
		**n (%)**	**n (%)**	**n (%)**	
**Gender** *	Female	362 (52.7)	2897 (48.7)	3259 (49.2)	3.578 (0.053)
	Male	325 (47.3)	325 (51.3)	3366 (50.8)	
Age**	Under 1 year	93 (13.6)	1630 (27.5)	1,723 (26.0)	96.3 (<0.0001)
	1–4 years	388 (56.6)	3266 (55.0)	3,654 (55.2)	
	5 years plus	205 (29.8)	1038 (17.5)	1,243 (18.8)	
**Education**	None	605 (88.1)	5407 (91.0)	6,012 (90.7)	
	Primary	56 (8.2)	394 (6.6)	450 (6.8)	7.698 (0.021)
	Secondary plus	26 (3.8)	140 (2.4)	166 (2.5)	
**Health Facility**	Bugiri District Hospital	44 (6.4)	637 (10.7)	681 (10.3)	85.5 (<0.0001)
	Gulu Regional Referral Hospital	107 (15.6)	1574 (26.5)	1681 (25.4)	
	Jinja Regional Referral Hospital	138 (20.1)	1228 (20.7)	1366 (20.6)	
	Kayunga District Hospital	11 (1.6)	158 (2.7)	169 (2.6)	
	Mulago National Referral Hospital	387 (56.3)	2344 (39.5)	2731 (41.2)	
**Total**		**687**	**5941**	**6628**	

Mean (SD) Age = 3.8 (7.0): Median (IQR) = 2 (2)

*3 missing gender and

**8 missing age

### Prevalence of Influenza by Surveillance Sites

Table 2 presents the prevalence of influenza at the sentinel surveillance sites. All 6,628 participants who fulfilled the ILI case definition provided samples for laboratory testing. Influenza virus was detected in 687 patients giving an overall influenza prevalence rate of 10.4%, where 468 (7.1%) patients were influenza A positive, 217 (3.3%) were influenza B positive and 2 (0.03%) patients had both influenza A and B. Mulago Hospital, the national referral and teaching hospital had the highest influenza prevalence of 14.2% with 387 of 2731 samples testing positive (September 2008 –December 2014). This was followed by Jinja Regional Referral Hospital with 138 (10.1%) samples positive (September 2009 –December 2014). The remaining three surveillance sites had a similar influenza prevalence of 6.4–6.5% during their respective surveillance duration.

**Table 2:** Period Prevalence of Influenza by Type and Surveillance Site 2008–2014.

**Sentinel Site**	**Total Samples Tested**	**Total Samples Positive** n **(%)**	**Influenza Positive**		
			Flu A n (%)	Flu B n (%)	Flu A and B n (%)
**Bugiri General Hospital**	681	44 (6.5)	32 (4.7)	11 (1.7)	1 (0.2)
**Gulu Regional Referral Hospital**	1681	107 (6.4)	70 (4.2)	36 (2.1)	1 (0.1)
**Jinja Regional Referral Hospital**	1366	138 (10.1)	94 (6.9)	44 (3.2)	0(0.0)
**Kayunga General Hospital**	169	11 (6.5)	6 (3.6)	5 (3.0)	0(0.0)
**Mulago National Referral Hospital**	2731	387 (14.2)	266 (9.7)	121 (4.4)	0(0.0)
**TOTAL**	**6628**	**687 (10.4)**	**468 (7.1)**	**217 (3.3)**	**2 (0.03)**

### Temporal and Seasonal Patterns of influenza

Table 3 presents the temporal distribution of the influenza type and subtype. Influenza prevalence was 11.5% in 2008, 16.7% in 2009 (highest), 7.3% in 2010 (lowest), 11.8% in 2011, 9.4% in 2012, 9.0% in 2013, and 8.1% in 2014. Out of the 687 samples which were influenza positive, 468 (68.1%) and 217 (31.6%) samples tested positive for influenza A and B, respectively, and 2 (0.03%) samples were co-infected with both influenza A and B viruses. A total of 470 samples containing influenza A (468 influenza A and 2 influenza A and B) were taken for sub-typing. The predominant sub-type was influenza A(H3N2) with 271/470 samples (57.7%); followed by influenza A(H1N1) pdm09 with 136/470 samples (28.9%), seasonal A (H1) with 9/470 samples (1.9%) and co-infection of influenza A(H3N2)/ influenza A(H1N1) pdm09 with 3/470 samples (0.6%). A total of 51 samples out of the 470 (10.9%) could not be subtyped.

**Table 3:** Distribution of type and subtypes of influenza virus in Uganda from October 2008 to December 2014.

	Year							
	2008	2009	2010	2011	2012	2013	2014	TOTAL
**Specimens tested**	365	831	1,419	876	863	1,172	1,102	6,628
**Flu A Positive**	42	78	94	68	68	27	91	468
**Flu B Positive**	0	61	9	35	11	78	23	217
**Flu A and B Positive**	0	0	0	0	2	0	0	2
Percentage of Positive (A OR B)**	11.5%	16.7%	7.3%	11.8%	9.4%	9.0%	8.1%	10.4%
**Subtypes of Flu A**								
**H1 (Seasonal)**	0	9	0	0	0	0	0	9
**H3N2**	42	20	58	21	62	9	59	271
**H3N2/Pandemic H1N1**	0	0	1	2	0	0	0	3
**Pandemic H1N1**	0	36	31	28	2	12	27	136
**Unsubtypeable**	0	13	4	17	6	6	5	51

** The only row with percentages and the rest are numbers

**Fig 2:**
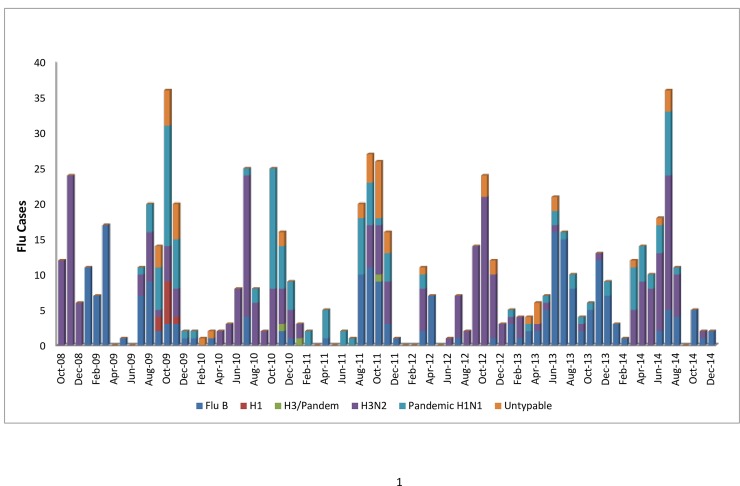
Temporal distribution of influenza type and subtype by month.

Seasonal influenza A(H3N2) was the only strain isolated in 2008 (Oct-Dec) and was the predominant strain in the years 2010, 2012, and 2014, while pandemic influenza A (H1N1) pdm09 was the predominant strain in 2009 and 2011. Pandemic influenza A(H1N1) pdm09 viruses became endemic and now co-exist with the seasonal influenza A(H3N2) strain. Between 2008 and 2014, influenza A viruses were dominant in all the years except for 2013 where influenza B was dominant. Notably, seasonal influenza A(H1N1) was observed in 2009 at a low level and was never detected again during this surveillance period; seasonal influenza A(H1N1) appears to be replaced by the pandemic influenza A(H1N1) pdm09 strain (Table 3). The distribution of influenza by month and year is presented in Fig 2. Influenza prevalence peaked in November during 2008, October during 2009, July and October during 2010, September during 2011, October during 2012, June during 2013, and July during 2014.

### Climatic factors and influenza patterns

Uganda is located on the equator with a tropical climate of moderate temperatures and two rainy seasons from March to May, and from October to December [21]. During the study time period, the five surveillance sites had an annual average rainfall ranging from 112.9 mm to 130.0 mm, an average temperature ranging from 22.7 to 24.8 degree Celsius, and an annual average relative humidity ranging from 61 to 73%.

**Fig 3:**
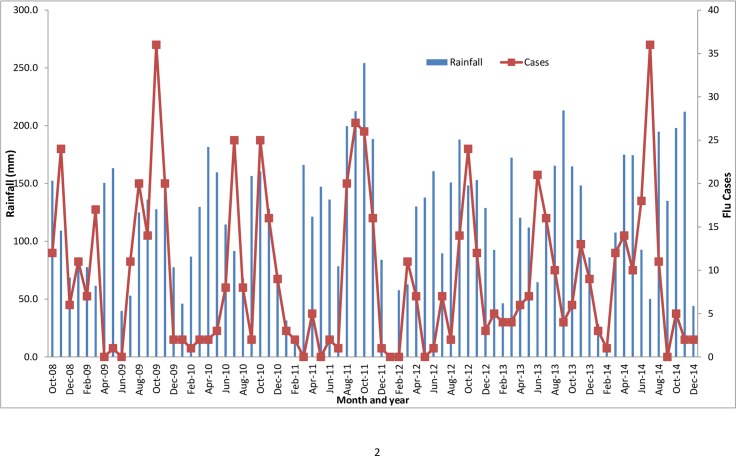
Rainfall and monthly number of flu cases.

**Fig 4:**
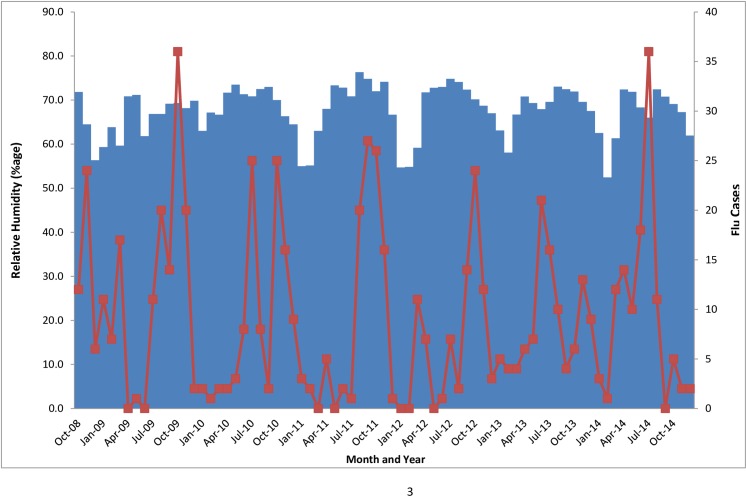
Humidity and monthly number of flu cases.

**Fig 5:**
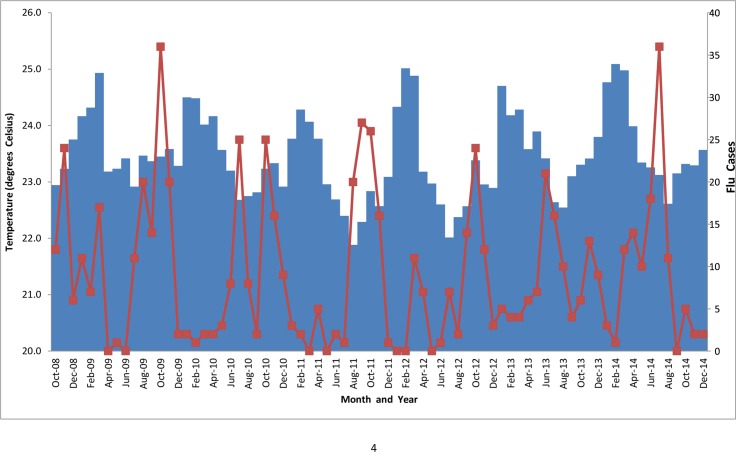
Average temperatures and monthly number of flu cases.

During this period, influenza infections in Uganda were observed all year round with peaks generally coinciding with months with higher rainfall and humidity and lower temperatures (Figs 3–5).

## Discussion

A clear understanding of the seasonality of influenza can inform prevention and clinical treatment strategies, such as which vaccines and when vaccination should be implemented to prevent major outbreaks. While the epidemiology of influenza is well characterized in temperate areas and developed countries, there is a paucity of relevant data in tropical areas, particularly sub-Saharan African countries. To address this gap, we analyzed influenza surveillance data in Uganda, a country with a tropical climate, from 2008 to 2014, and explored the relationship of influenza burden with seasonality. Our observation that influenza is present in Uganda throughout the year is consistent with other studies [22] Further, our finding that influenza peaks mainly during the rainy season when the relative humidity is high and temperatures are low is also consistent with other studies that have examined the role of tropical climatic factors on influenza [23, 24].

### Viruses identified

The predominance of influenza types varied from year to year. Influenza A virus was the main type throughout the study time period, except when influenza B was the main type in 2013. The predominance of influenza A virus sub-types also varied over time. Seasonal influenza A (H1N1) was only isolated in 2009, influenza A (H1N1) pdm09 was the dominant sub-type in 2009, 2011, and 2013, and seasonal influenza A (H3N2) was the dominant sub-type all other years. While seasonal influenza A (H3N2) dominated the sub-types, the pandemic strain of H1N1 became endemic and circulated with other seasonal influenza. The trends of influenza A sub-types observed in this study are similar to trends described in an earlier study in Uganda [2].

### Demographic characteristics of this population compared to others

Our finding that children less than 5 years had the highest incidence of ILI is consistent with findings from other studies in sub-Saharan Africa [25] and Bangladesh [26]. Generally, younger children are more susceptible to influenza viral transmission and therefore they present with a higher incidence than other age groups [27, 28]. However, it is also possible that low numbers of ILI observed among older children and adults is a reflection of the health-seeking behaviour of these age groups: that older children and adults with ILI symptoms do not visit a healthcare provider unless they develop severe symptoms that cannot be treated with self-medication. Also, it is possible to older people may take medicine to bring down the fever before visiting the health facility and they are excluded from the study because they do not fill the temperature requirements of an ILI case definition.

### Seasonal and temporal variation in occurrence of influenza

Uganda lies astride the equator and is therefore affected by the migration of the Intertropical Convergence Zone (ITCZ), resulting in two distinct wet and dry seasons for the country [29]. The seasonal patterns in temperate regions tend to be more consistent; in contrast, the wet and dry seasons in tropical countries near the equator do not always fall on the same months each year [30]. In general, Uganda receives the most rain between March and June, and September and November, and the country has the least amount of rainfall from December to February, and June to August [31]. In our study, the most number of influenza cases occur in the months from August to November, which coincides generally with the longest rainy season in Uganda. This finding is consistent with other findings from the Ivory Coast and Ghana that influenza cases increase with increased periods of rainfall, high humidity, and low temperatures in tropical countries [24, 32, 33].

### Study Limitations

While we tried to capture ILI cases in patients of all ages in order to identify the circulating strains at the hospital-based sentinel sites, the study is disproportionately represented by children less than 5 years. It is possible that influenza strains common to older children and adults are not identified because of selection bias resulting from lesser health-seeking for ILI among older children and adults compared to young children less than 5 years.

## Conclusions

This is the first study in Uganda to describe the epidemiology of influenza using long-term surveillance data from outpatient departments of hospital-based sentinel sites. Our findings on influenza circulating all year-round with peaks corresponding to the two rainy seasons in the region are consistent with other research, and importantly, contributes evidence to our growing understanding of the relationship between seasonality and influenza in temperate regions of sub-Saharan Africa. Clarifying what influenza strains are circulating and the patterns of their occurrence are critical to improving the country’s routine preparedness against influenza outbreaks. Further, it is important for national governments and partners to continue surveillance efforts for rapid detection of unusual influenza activity or strains that can cause pandemic outbreaks.

## Supporting Information

S1 File Uganda Influenza Paper Dataset. (DTA)

S2 File Uganda Climate Data. (XLS)
